# Genome-guided prediction of acid resistance mechanisms in acidophilic methanotrophs of phylogenetically deep-rooted Verrucomicrobia isolated from geothermal environments

**DOI:** 10.3389/fmicb.2022.900531

**Published:** 2022-09-23

**Authors:** Gonzalo Neira, Eva Vergara, David S. Holmes

**Affiliations:** ^1^Center for Bioinformatics and Genome Biology, Centro Ciencia & Vida, Fundación Ciencia & Vida, Santiago, Chile; ^2^Facultad de Medicina y Ciencia, Universidad San Sebastián, Santiago, Chile

**Keywords:** geothermal ecosystems, acid resistance mechanisms in hyperacidic environments, polyextremophiles, *Verrucomicrobia* phylogenomics, methanotrophy, lanthanides, horizontal gene transfer (HGT), biomining

## Abstract

Verrucomicrobia are a group of microorganisms that have been proposed to be deeply rooted in the Tree of Life. Some are methanotrophs that oxidize the potent greenhouse gas methane and are thus important in decreasing atmospheric concentrations of the gas, potentially ameliorating climate change. They are widespread in various environments including soil and fresh or marine waters. Recently, a clade of extremely acidophilic Verrucomicrobia, flourishing at pH < 3, were described from high-temperature geothermal ecosystems. This novel group could be of interest for studies about the emergence of life on Earth and to astrobiologists as homologs for possible extraterrestrial life. In this paper, we describe predicted mechanisms for survival of this clade at low pH and suggest its possible evolutionary trajectory from an inferred neutrophilic ancestor. Extreme acidophiles are defined as organisms that thrive in extremely low pH environments (≤ pH 3). Many are polyextremophiles facing high temperatures and high salt as well as low pH. They are important to study for both providing fundamental insights into biological mechanisms of survival and evolution in such extreme environments and for understanding their roles in biotechnological applications such as industrial mineral recovery (bioleaching) and mitigation of acid mine drainage. They are also, potentially, a rich source of novel genes and pathways for the genetic engineering of microbial strains. Acidophiles of the *Verrucomicrobia* phylum are unique as they are the only known aerobic methanotrophs that can grow optimally under acidic (pH 2–3) and moderately thermophilic conditions (50–60°C). Three moderately thermophilic genera, namely *Methylacidiphilum, Methylacidimicrobium*, and Ca. *Methylacidithermus*, have been described in geothermal environments. Most of the investigations of these organisms have focused on their methane oxidizing capabilities (methanotrophy) and use of lanthanides as a protein cofactor, with no extensive study that sheds light on the mechanisms that they use to flourish at extremely low pH. In this paper, we extend the phylogenetic description of this group of acidophiles using whole genome information and we identify several mechanisms, potentially involved in acid resistance, including “first line of defense” mechanisms that impede the entry of protons into the cell. These include the presence of membrane-associated hopanoids, multiple copies of the outer membrane protein (Slp), and inner membrane potassium channels (*kup, kdp*) that generate a reversed membrane potential repelling the intrusion of protons. Acidophilic Verrucomicrobia also display a wide array of proteins potentially involved in the “second line of defense” where protons that evaded the first line of defense and entered the cell are expelled or neutralized, such as the glutamate decarboxylation (*gad*AB) and phosphate-uptake systems. An exclusive N-type ATPase F_0_-F_1_ was identified only in acidophiles of Verrucomicrobia and is predicted to be a specific adaptation in these organisms. Phylogenetic analyses suggest that many predicted mechanisms are evolutionarily conserved and most likely entered the acidophilic lineage of Verrucomicrobia by vertical descent from a common ancestor. However, it is likely that some defense mechanisms such as *gad*A and *kup* entered the acidophilic Verrucomicrobia lineage by horizontal gene transfer.

## Introduction

Acidophiles can be defined as organisms that grow optimally at pH <5 (Dopson, 2016). A common further classification defines moderate acidophiles as organisms that grow optimally between pH 3 and 5 (Foster, 2004; Benison et al., 2021) and extreme acidophiles that grow optimally at pH ≤ 3 (Johnson and Aguilera, 2015). The latter are particularly challenged for survival as they tolerate proton gradients across their membrane several orders of magnitudes higher than neutrophiles or moderate acidophiles (Baker-Austin and Dopson, 2007; Slonczewski et al., 2009; Quatrini and Johnson, 2018; Hu et al., 2020). Acidophiles are found in anthropogenic environments such as industrial mineral recovery (bioleaching) sites and acid mine drainage (AMD) and in natural environments including acidic hot springs, volcanic mud pools, and acid rock drainage (ARD) (Baker-Austin and Dopson, 2007; Huang et al., 2013; Cárdenas et al., 2016; Schmitz et al., 2021). Acidophilic organisms are important in the biotechnology industry as they play key roles in biomining processes and bioremediation of AMD (Johnson and Hallberg, 2005; Schippers et al., 2013). They are also a source of acid-stable enzymes (Baker-Austin and Dopson, 2007; Sharma et al., 2012; Dopson and Holmes, 2014; Liu et al., 2015). Furthermore, they are important from an evolutionary standpoint as some members inhabit what are thought to be early Earth-like environments (González-Toril et al., 2005; Baker-Austin and Dopson, 2007; Mirete et al., 2017). Extreme acidophiles are found across all the Tree of Life (ToL) with members in Bacteria, Archaea, and Eukarya domains (Hedrich and Schippers, 2021).

The phylum Verrucomicrobia is composed mainly of neutrophiles found commonly in soil and fresh or marine waters (Bergmann et al., 2011; Freitas et al., 2012; Cabello-Yeves et al., 2017) and also as part of the intestinal microbiome (de Vos, 2017). Exceptionally, a clade of extremely acidophilic aerobic methanotrophs has been described in geothermal environments (Dunfield et al., 2007; Pol et al., 2007; Islam et al., 2008; van Teeseling et al., 2014). This clade includes organisms from three different genera: *Methylacidiphilum*, identified in hot acidic geothermal environments (optimal growth pH 2 - 3 and optimal temperature growth 50–60 °C) (Hou et al., 2008; Islam et al., 2008; Anvar et al., 2014); *Methylacidimicrobium*, from the same geothermal location but living at lower temperatures (optimal growth temperature 30 to 44°C and optimal growth pH between 1 and 3) (van Teeseling et al., 2014) with the exception of *Methylacidimicrobium thermophilum* AP8 which grows optimally at 50 °C and pH 3 to 5 (Picone et al., 2021a). A third organism, Ca. *Methylacidithermus pantelleriae*, from a metagenome-assembled genome (MAG) represents the only known member of this proposed genus (Picone et al., 2021b). All three genera are characterized by being the only bacteria capable of oxidizing methane (methanotrophy) in extremely acidic environments (van Teeseling et al., 2014). Recently, it has also been discovered that organisms from these genera can grow as mixotrophs by oxidation of H_2_, in addition to methane, as an adaptation to the fluctuations of methane in the geothermal environments they inhabit (Carere et al., 2017; Mohammadi et al., 2017, 2019; Schmitz et al., 2021). They also use lanthanides instead of other metals as co-factors in some enzymes (Pol et al., 2014) and are being exploited for the development of industrial applications of rare earth elements including lanthanide (Cotruvo, 2019; Featherston and Cotruvo, 2021).

Phylogenetic profiles of Verrucomicrobia acidophiles have been based on analyses using 16S rRNA (Dunfield et al., 2007; Pol et al., 2007; van Teeseling et al., 2014; Picone et al., 2021b; Schmitz et al., 2021), *pmoA* (Anvar et al., 2014; Picone et al., 2021a), or seven housekeeping genes (Erikstad et al., 2019). In this paper, we extend the phylogenetic analyses of this group using fully sequenced genomic data, including all sequenced genomes of Verrucomicrobia methanotrophs.

To date, most of the extensive studies of extreme acid resistance and its evolution have been made in organisms that aerobically oxidize reduced iron and/or sulfur in biomining operations and similar acidic econiches (e.g., AMD) (Baker and Banfield, 2003; Valdés et al., 2008; Cárdenas et al., 2016; Christel et al., 2018; Vergara et al., 2020; Zhang et al., 2020b; González-Rosales et al., 2022). These organisms maintain a near neutral pH inside the cell while confronting an external pH that can be several orders of magnitude lower (e.g., pH ≤ 3) (Baker-Austin and Dopson, 2007). Acid resistance mechanisms have been classified for convenience of discussion into two different classes, a “first line” of defense, which encompasses membrane changes designed to impede the entry of protons into the cell, and a “second line” of defense, which either consumes or expels protons that have penetrated the cytoplasmic membrane (Vergara et al., 2020).

Previous studies have identified genes predicted to form part of the “first line” of defense in the moderate thermophilic group *Methylacidiphilum* (Hou et al., 2008; Kruse et al., 2019). Six different copies of the starvation lipoprotein (Slp), a protein with no proven function but that is co-expressed in an acid resistance island in *E. coli* (Mates et al., 2007) were identified in *M. infernorum* V4 and five different orthologs also identified in both other complete genomes *M. fumariolicum* SoIV and *M. kamchatkense* Kam1. Also, two potassium transporters, Kch and Kdp, were described which have been shown in other microorganisms to generate an inner membrane positive charge that repels protons (Buetti-Dinh et al., 2016; Christel et al., 2018). In addition, modification of the fluidity of the membrane *via* the incorporation of hopanoids has also been proposed to be present in a wide range of acidophiles and deletion of the hopanoid biosynthesis pathway in acid-tolerant bacteria reduces the growth at low pH (Welander et al., 2009; Mangold et al., 2013; Sohlenkamp and Geiger, 2016), but this has not yet been investigated in the acidophilic Verrucomicrobia.

Mechanisms, potentially forming part of the second line of defense against acid resistance, have been reported previously in some strains of *Methylacidiphilum* but are unexplored in *Methylacidimicrobium* and *Methylacidithermus* (Hou et al., 2008; Kruse et al., 2019). Amino acid decarboxylation systems, such as arginine and glutamate decarboxylation, include enzymes that work in pairs with the corresponding transporter by binding protons and then secreting them outside the membrane (Foster, 2004). Another second line of defense mechanism involves proton/cation antiporters (e.g., sodium or calcium cations) that belong to a large family of transporters that can regulate the intracellular pH by the expulsion of protons while maintaining the cytoplasmic charge unchanged by the entrance of a positively charged cation (Padan et al., 2004). The ATPase F_0_-F_1_ is a common mechanism that can work either by importing protons to generate ATP or in a reverse fashion resulting in the expulsion of the protons (Foster, 2004).

In this study, we expand our knowledge of potential acid resistance systems in strains of the extremely acidophilic genera, *Methylacidiphilum, Methylacidimicrobium*, and Ca. *Methylacidithermus*. Predicted acid resistance genes and pathways were then used to build a global model of pH homeostasis mechanisms in these genera that allowed us to hypothesize how acid resistance was acquired during the evolution of acidophilic methanotrophs. It is hypothesized that vertical inheritance from a common ancestor that is predicted to be a neutrophile and Horizontal Gene Transfer (HGT) from other acidophiles are the main mechanisms for the acquisition of acid resistance in acidophilic methanotrophs.

## Materials and methods

### Retrieval of genome sequences and quality assessment

A dataset of 20 genome sequences from acidophilic methanotrophs of the Verrucomicrobia phylum were obtained from AciDB (Neira et al., 2020) and retrieved using the National Center for Biotechnology Information (NCBI) assembly ID through the Batch Entrez tool (Kans, 2020). In addition, 49 genomes of neutrophiles classified as representative species in the genome taxonomy database (GTDB) (Parks et al., 2022) and NCBI were retrieved using the Batch Entrez tool. From these genomes, 24 are associated with the phylum Planctomycetes and were used exclusively for phylogenetic analysis and 25 from the Verrucomicrobia phylum were used for both phylogenetic and comparative genomics analysis. Quality assessment of all genomes was carried out by CheckM v1.1.2 (Parks et al., 2015), defining >90% completeness and <10% contamination as high-quality genomes according to the proposed standards for high-quality draft genomes (Chun et al., 2018). Detailed information on the nomenclature, taxonomy, and source of the genomes in our study is provided in Supplementary Table 1.

### Genome characterization

Average nucleotide identity (ANI) between genomes was calculated with FastANI v1.1 using default parameters, where organisms with ANI values >95% were considered to belong to the same species (Jain et al., 2018). In a group of species clustered together with ANI > 99%, only the highest quality genome was selected for subsequent analysis. Digital DNA-DNA hybridization (dDDH) was carried out using the genome-to-genome distance calculator v2.1 (GGDC) (Meier-Kolthoff et al., 2013) with Formula 2 as recommended for datasets with draft genomes. Average amino acid identity (AAI) was calculated between each pair of genomes using CompareM (https://github.com/dparks1134/CompareM), using default parameters.

### Phylogenomic analyses

Phylogenetic reconstruction was inferred using the PhyloPhlAn3 pipeline using a set of 400 universal markers (Asnicar et al., 2020). The parameters utilized in each of the steps of the PhyloPhlAn3 pipeline are the following: Diamond v2.0.11 (Buchfink et al., 2015) was used for mapping each marker in the different genomes; MAFFT (Katoh et al., 2005) with the L-INS-I algorithm for the multiple sequence alignment (MSA) of identified markers; IQTREE v1.6.12 (Nguyen et al., 2015) was used for the construction of maximum-likelihood trees from the concatenated MSA; Modelfinder (Kalyaanamoorthy et al., 2017) was used to select the best-suited evolutionary models according to the Bayesian (BIC) and Akaike information criterion (AIC); and UFBoot (Minh et al., 2013) was used to infer the robustness of the tree with 1,000 replicates. Domain composition of proteins was predicted using used PFAM domains in TREND (Gumerov and Zhulin, 2020, 2022). The consensus tree was visualized using iTOL v6 (Letunic and Bork, 2019) and edited with Inkscape (https://inkscape.org/).

### Identification of orthologous groups of genes

Orthogroups are defined as proteins that have evolved from a common ancestor and include both orthologs (homologous proteins from two species) and paralogs (set of proteins that have a common origin in the same genome) and were identified using Orthofinder v2.3.3 (Emms and Kelly, 2018). Predicted protein sequences were compared using Diamond v2.0.11 (Buchfink et al., 2015) in an all-versus-all sequence similarity search. DendroBLAST (Kelly and Maini, 2013) was used to generate unrooted protein trees for each of the identified orthogroups. Protein alignment for all orthogroups was performed using MAFFT (Katoh et al., 2005). FastTree (Price et al., 2010) was used for tree inference in each orthogroup.

### Acquisition and analyses of acid resistance genes

Potential genes and pathways involved in microbial resistance against low pH were identified through an extensive literature search and were functionally characterized using Pfam (Finn et al., 2014) and InterPro families (Blum et al., 2021), providing a curated list of proteins associated with acid resistance (Supplementary Table 2). All genomes were functionally annotated using the Interproscan pipeline (Jones et al., 2014). InterPro and Pfam were then used to identify similar proteins in the genomes of acidophiles and neutrophiles of Verrucomicrobia using a custom python script. All proteins predicted to be involved in acid resistance were identified in the previously defined orthogroups in order to analyze the conservation across the organisms in the study (Supplementary Table 3). The conservation of genetic context between Verrucomicrobia genomes and manually curated mis-annotations for the genes of interest were determined by MAUVE (Darling et al., 2004). Phylogenetic tree reconstructions were made as described above for all orthogroups involved in acid resistance.

### Evolutionary inference of acid resistance genes

Inference of branch-site-specific events was made using the tree topology of the 400 conserved markers species tree obtained in the phylogenomic analysis as described above. The presence and absence of genes related to acid resistance and associated genes (same genomic context) were mapped onto each branch of the phylogenetic tree. The inference of evolutionary events was made using maximum parsimony criteria as incorporated in the COUNT software (Csuös, 2010). HGT predictions were made using HGTector (Zhu et al., 2014). To study in more detail, possible HGT events phylogenetic trees were constructed for each predicted protein to be acquired by these mechanisms, and in these cases, the 100 best hits from BLASTP against the RefSeq protein database followed by removal of duplicate sequences using SeqKit (Shen et al., 2016) were added to the sequences in the corresponding orthogroup. Phylogenetic tree construction was performed with the same parameters and software detailed in the previous section with the additional step for the estimation of the root position for each orthologous unrooted tree with minimal ancestor deviation (MAD) method applied with MAD phylogenetic rooting algorithm (Tria et al., 2017).

## Results and discussion

### Genomic index and features

Twenty genome sequences from acidophiles of the bacterial phylum Verrucomicrobia were downloaded, of which 7 are complete genomes and 13 are in draft state. All genomes have a contamination index lower than 10% and a completeness index higher than 90% as defined by CheckM. Acidophiles and neutrophiles genome sizes showed differences; the acidophilic clade has an average genome size of 2.37 Mb, in contrast to neutrophiles clades of *Opitutales, Chtoniobacterales*, and *Verrucomicrobiales* having an average size of 5.34, 6.3, and 4.28 Mb, respectively (Cortez et al., 2022) (Supplementary Table 1). Genomic indices were calculated to identify the taxonomic rank and species for the genomes under study. ANI and dDDH values were used to evaluate the species clusters in the dataset; in addition, ANI values over 99% were used to de-replicate the genomes for the subsequent analysis (values summarized in Supplementary Tables 4, 5, respectively). Species clusters not previously reported were identified in the case of *Methylacidiphilum* sp. IT5 with *Methylacidiphilum infernorum* (ANI of 96% and dDDH of 68.7 using formula 2 with a 75% probability of both genomes being the same species) and *Methylacidiphilum* sp. IT6 with *Methylacidiphilum* sp. Phi (ANI of 98% and dDDH of 86). All previously reported species were also supported by this analysis. Five genomes from *Methylacidiphilum fumariolicum* and one genome of *Methylacidiphilum kamchatkense* were excluded for further analysis by de-replication criteria, and in both cases, the complete genome of each species was kept. AAI values were used to analyze the genus-level taxonomic association of the genomes (Supplementary Table 6). The values divide the different species into three different genera: (i) Ca. *Methylacidithermus pantelleriae* is the only representative for *Methylacidithermus*; (ii) *Methylacidimicrobium* with six different species, and (iii) *Methylacidiphilum* with five different species. An overview of the final 14 genomes with their genomic characteristics and geographic origin is presented in Table 1.

**Table 1:** Genomic features of the organisms used in this study.

**Genome**	**Size (Mb)**	**# Predicted genes**	**G+C (%)**	**pH**	**Temp (°C)**	**Status^1^**	**NCBI accession**	**Geographical origin**	**Refs**
*Ca. Methylacidithermus pantelleriae*	2.46	2,214	55.2	4–4.5	60-90	D	GCA_905250085.1	Pantelleria Island, Italy	Picone et al., 2021b
*Methylacidimicrobium cyclopophantes*	2.28	2,217	61.2	1.5–3	44	D	GCA_902143385.2	Solfatara crater, Naples, Italy	van Teeseling et al., 2014
*Methylacidimicrobium tartarophylax*	2.33	2,241	61.2	1–3	38	D	GCA_902143375.2	Solfatara crater, Naples, Italy	van Teeseling et al., 2014
*Verrucomicrobium sp*. 3C	2.77	2,456	60.8	1.5–3	35	D	GCA_000379365.1	Solfatara crater, Naples, Italy	van Teeseling et al., 2014
*Methylacidimicrobium sp*. AP8	2.3	2,361	64.3	3–5	50	C	GCA_903064525.1	Favara Grande, Pantelleria Island, Italy	Picone et al., 2021a
*Verrucomicrobia bacterium* LP2A	2.47	2,391	62.7	3.1	30	D	GCA_000526255.1	Waikato, New Zealand	Sharp et al., 2014
*Methylacidimicrobium sp*. B4	2.37	2,221	63.6	4.5	42	C	GCA_017310545.1	Pozzuoli, Italy	Awala et al., 2021
*Methylacidiphilum sp*. Yel	2.25	2,151	41.1	2.5–3.5	53–57	D	GCA_004421185.1	Yellowstone National Park, United States	Erikstad et al., 2019
*Methylacidiphilum fumariolicum* SolV	2.48	2,077	41.5	2	55	C	GCA_000953475.1	Campi Flegrei, Italy	Anvar et al., 2014
*Methylacidiphilum kamchatkense* Kam1	2.2	2,018	40.3	2–3.5	55–60	C	GCA_007475525.1	Kamchatka, Russia	Kruse et al., 2019
*Methylacidiphilum sp*. IT5	2.19	2,016	44.5	4.5	55	C	GCA_017310525.1	Pozzuoli, Italy	Awala et al., 2021
*Methylacidiphilum infernorum* V4	2.29	2,113	45.5	2–2.5	60	C	GCA_000019665.1	Tikitere, New Zealand	Hou et al., 2008
*Methylacidiphilum sp*. IT6	2.25	2,079	40.7	4.5	55	C	GCA_017310505.1	Pozzuoli, Italy	Awala et al., 2021
*Methylacidiphilum sp*. Phi	2.34	2,259	41.4	2.5–3.5	53–57	D	GCA_004421255.1	Makiling mud spring, Philippines	Erikstad et al., 2019

1 D, Draft genomes; C. Complete genomes.

### Phylogenomic analysis

**Figure 1:**
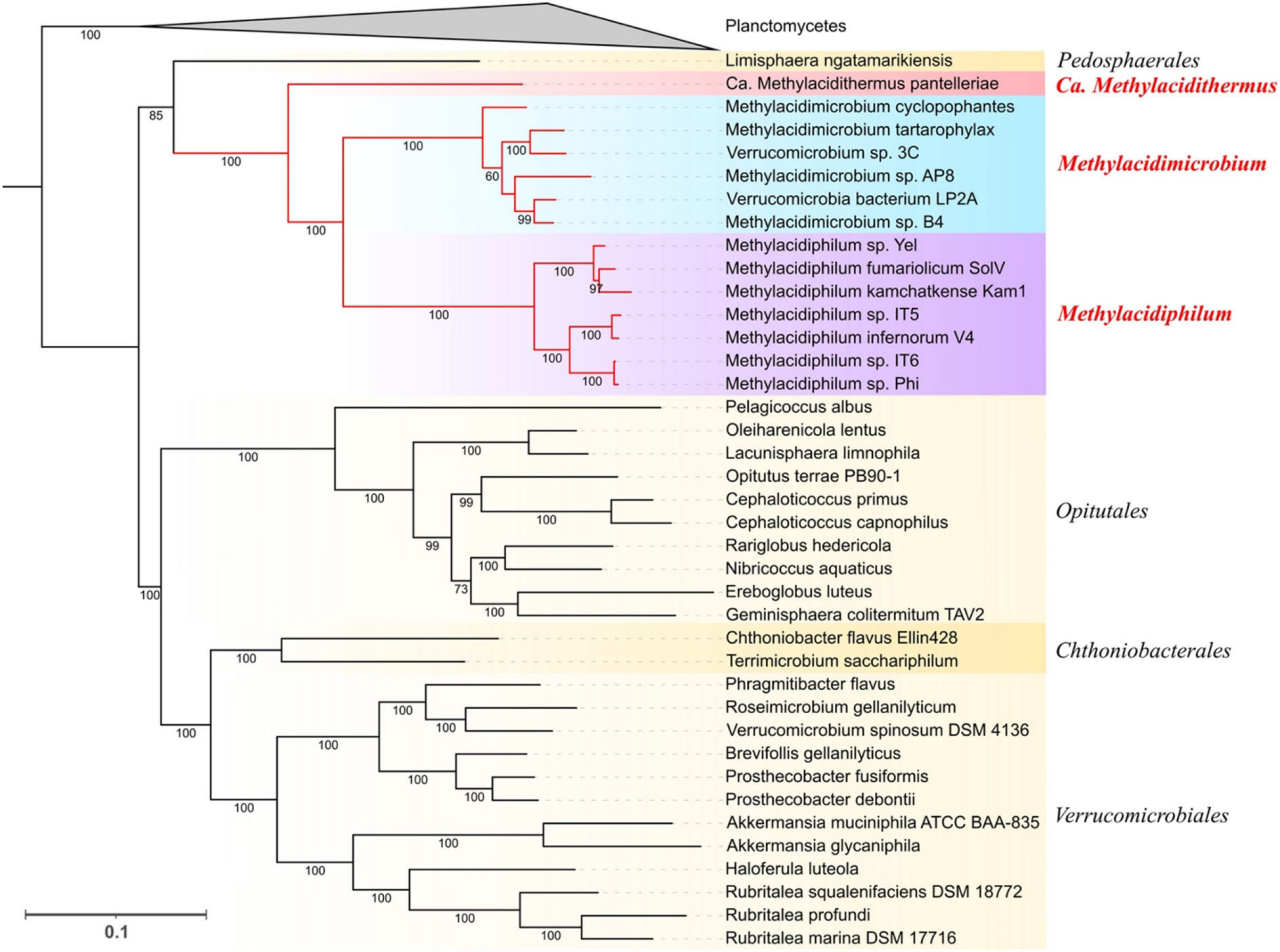
Phylogenetic distribution of Verrucomicrobia. Maximum Likelihood tree obtained with PhyloPhlAn3 using 400 conserved markers. Planctomycetes genomes were collapsed and used as an outgroup. Support values of the ultrafast bootstrap repetitions are shown in each corresponding branch. The taxonomic rank of each clade is shown at the right of the tree, genus level for all the acidophilic genomes, and order level for the neutrophiles (in yellow shadow).

A phylogenomic tree was reconstructed based on the 400 conserved markers identified with PhyloPhlAn3 resulting in an alignment of 9,640 positions (Figure 1). The tree was rooted using genomes from the Planctomycetes phylum as an outgroup. Two main clades are distinguished, the first one formed by acidophilic genomes from all three genera and *Limisphaera ngatamarikiensis* (only representative genome of the *Pedosphaerales* order) and the second one with all neutrophiles from the orders *Opitutales, Chthonobacteriales*, and *Verrucomicrobiales*. The branching order in the acidophilic clade suggests that Ca. *Methylacidithermus pantelleriae* is the first to branch followed by *Methylacidimicrobium* and *Methylacidiphilum*. This genome-driven phylogeny supports the branching order reported for a 16S rRNA tree (Schmitz et al., 2021) and consolidates and expands previous phylogenomic studies that used gene markers (Cai et al., 2022; Kim et al., 2022).

### Acid resistance: First line of defense

Proton influx into the cell is thought to be reduced in acidophiles by the development of an inside positive potential which is different from neutrophiles that have inside negative potential (Richard and Foster, 2004; Baker-Austin and Dopson, 2007). One of the ways that acidophiles generate this positive potential is *via* the use of membrane cation transporters, especially K^+^ transporters. Acidophiles also have a highly impermeable cell membrane to restrict proton influx into the cytoplasm (Konings et al., 2002) that can be generated by altering the lipid and fatty acid composition of the membrane (Feng et al., 2021). Hopanoid incorporation into the membrane has also been implicated in enhancing membrane stability (Welander et al., 2009).

#### Potassium uptake systems

**Figure 2:**
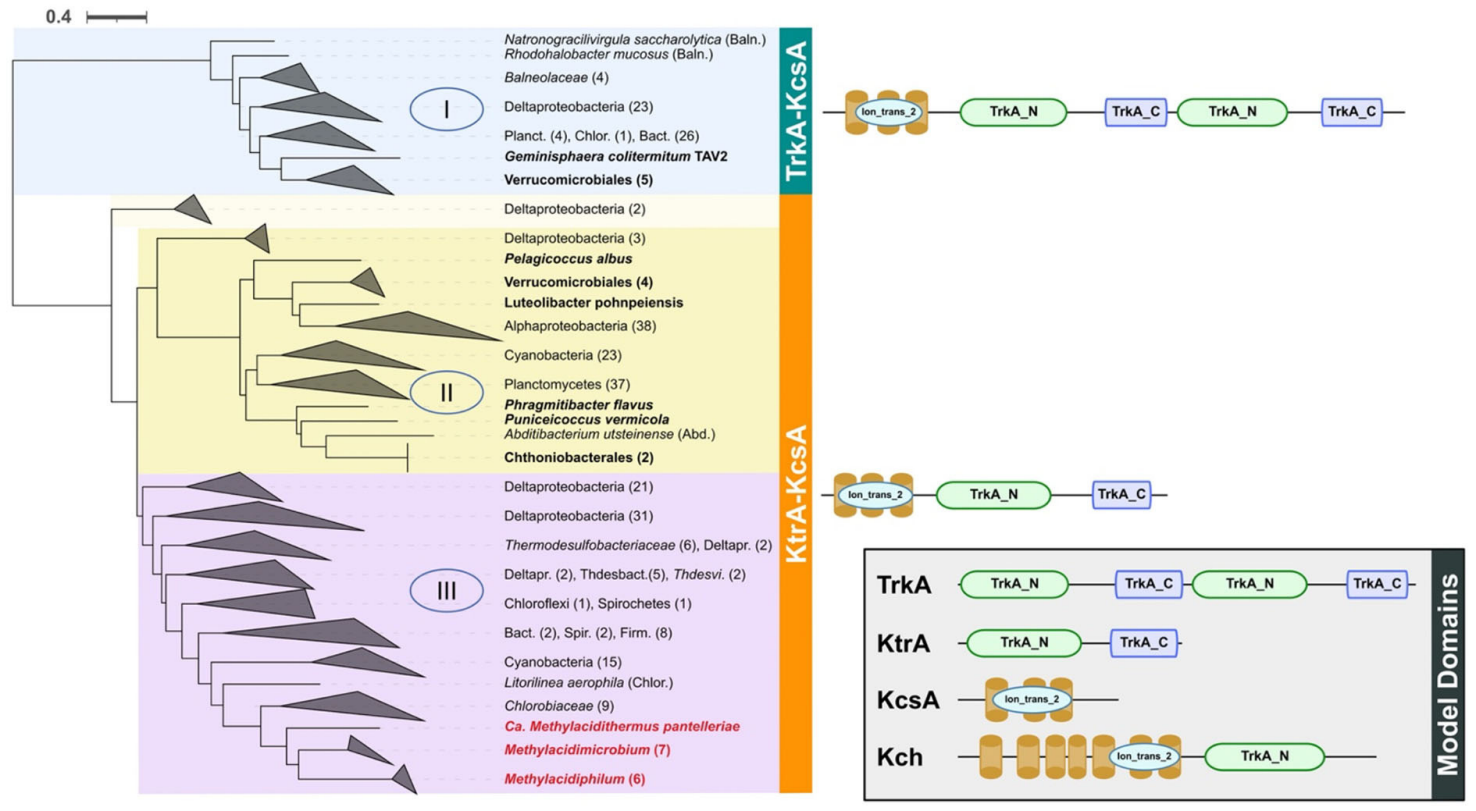
Phylogenetic tree and domain composition of potassium transporter vCh. In the blue background is shown clade I, yellow = clade II, and purple = clade III. All sequences were clustered to phylum or class level when possible. Acidophiles are indicated in red letters and the Verrucomicrobia neutrophiles are in bold letters. On the right side is shown the domain composition of proteins in each clade as predicted by PFAM in TREND, where ion_trans_2 = domain responsible for recognition of K+. At the right bottom of a box are the Model Domains for the different potassium channels (Uniprot accession as reference); TrkA from *Escherichia coli* (acc: P0AGI8) and *Salmonella typhimurium* (acc: P0A2J9), Ktr from *Bacillus subtilis* (acc: O32080), KcsA from *Streptomyces lividans* (acc: P0A334), and Kch from *E. coli* (acc: P31069). Baln, Balneolaceae; Planc, Planctomycetes; Chlor, Chloroflexi; Bact, Bacteroidetes; Abd, Abditibacteriota; Deltapr, Deltaproteobacteria; Thdesbact, Thermodesulfobacteriales; Thdesvi, Thermodesulfovibrio; Spir, Spirochetes; Firm, Firmicutes.

**Figure 3:**
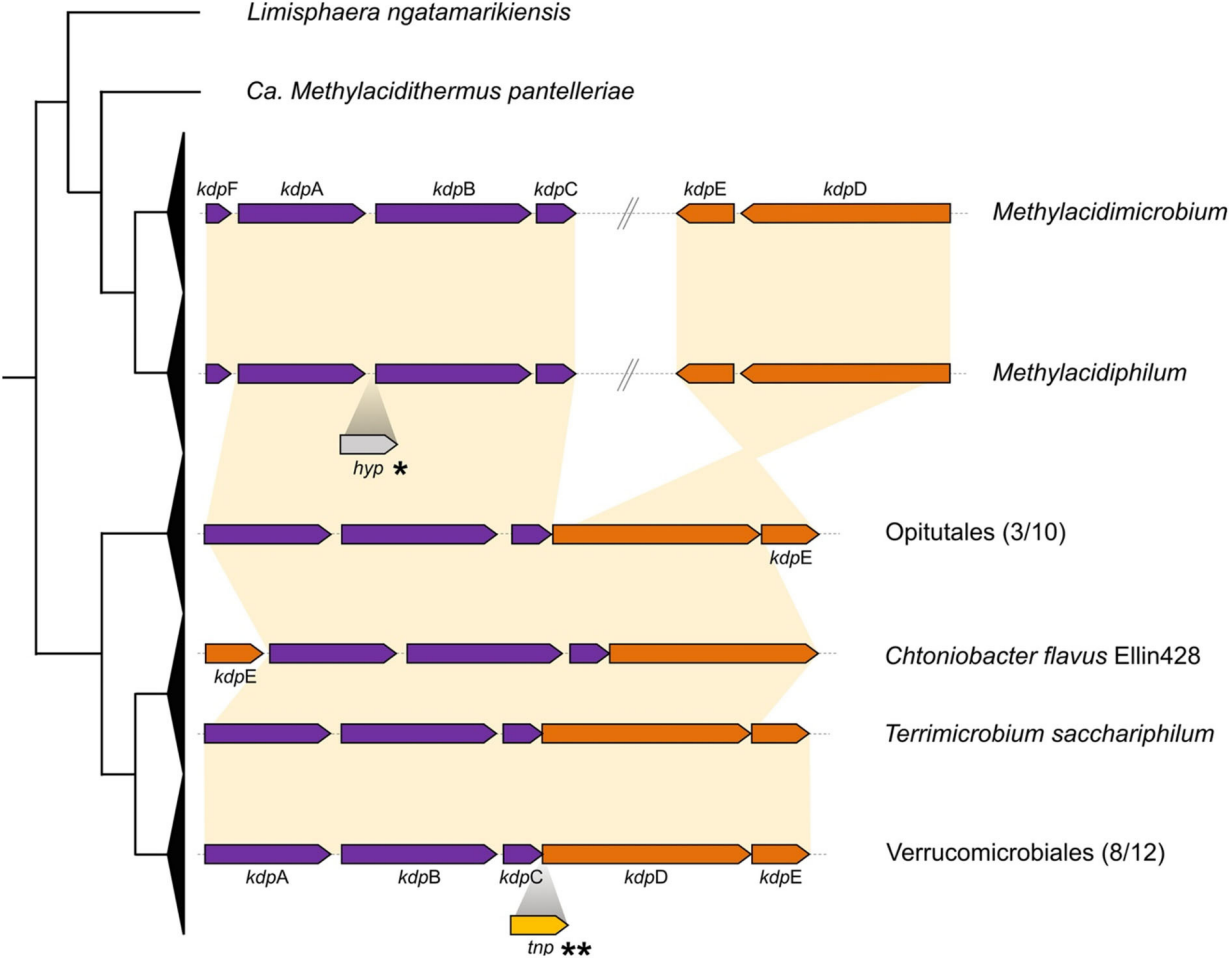
Cluster organization for kdp genes. Collapsed cladogram of Verrucomicrobia is represented on the left. Functional genes are shown in purple and regulator subunits are shown in orange, with synteny represented by yellow connecting background. *Insertion of hypothetical protein identified in *Methylacidiphilum kamchatkense*. **Transposase identified in *Phragmitibacter flavus* and *Brevifollis gellanilyticus*.

**Figure 4:**
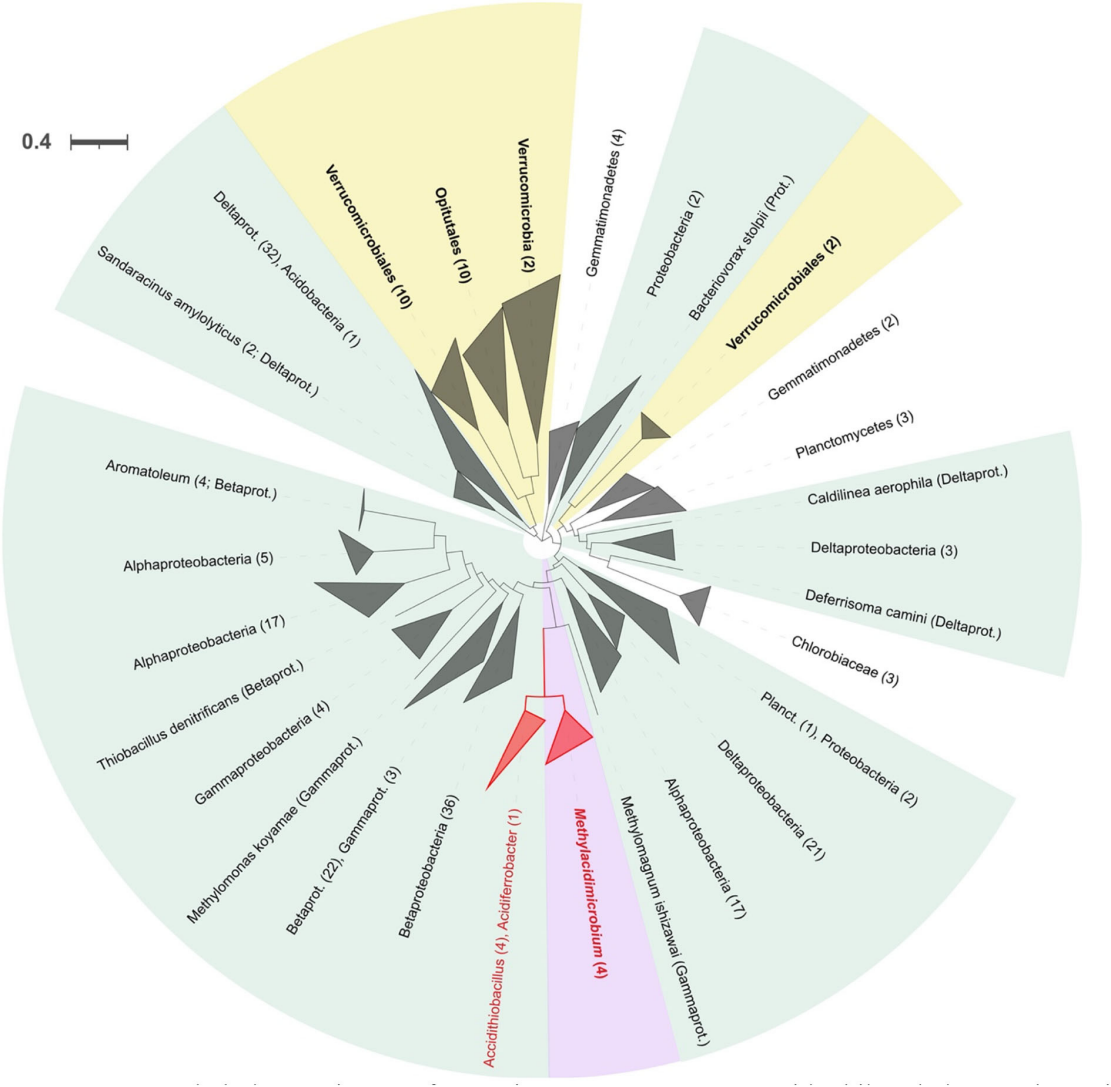
Rooted phylogenetic tree of potassium transporter Kup. Acidophiles clades are shown in red with violet background for acidophiles from Verrucomicrobia. Yellow background and bold letters for neutrophilic Verrucomicrobia, green background for neutrophiles from Proteobacteria, and other phyla are without color background.

Three different potassium uptake systems were identified in the methanotrophs:

(i) a voltage-associated potassium channel (vCh) was predicted in all acidophiles and in 8 of 25 neutrophiles. A phylogenetic tree was constructed with vCh and rooted with MAD (as described in Methods). Three main clades of vCh (clades I, II, and III, Figure 2) were identified, all sharing a common architecture with similarity to the potassium transporter KcsA; the latter could be a potential starting point in the evolution of potassium channels (Diskowski et al., 2015). Domain composition analysis (Zhang et al., 2020a) of clade I that contains sequences from *Verrucomicrobiales* and *Opitutales* suggests that it evolved as a fusion between TrkA and KcsA. Clades II and III share a similar architecture with an N-terminal KcsA-like domain and a C-terminal KtrA-like domain (Figure 2). Clade II contains *Verrucomicrobiales* that branch close to Planctomycetes, Cyanobacteria, and Alphaproteobacteria. Clade III contains all the acidophilic methanotrophs in a monophyletic branch that branches as the final “twig” from a group that contains Deltaproteobacteria, Cyanobacteria, and *Chlorobiaceae*. The diversity in phylum and families forming the clade III, out from Verrucomicrobia, strength the HGT possibility for this gene in the acidophiles. The last is supported by additional positive results for the *Methylacidiphilum* from HGTector that suggests this gene could be related to an HGT event (Supplementary Table 7).
(ii) A second potassium uptake system identified is the high-affinity system Kdp (Epstein, 2003). The classic composition of the Kdp cluster is characterized by the presence of the effector subunits KdpFABC, in charge of forming the inner membrane potassium transporter and the two-component regulatory system KdpDE. The gene cluster formed by *kdpABC* was identified in *Methylacidimicrobium* and *Methylacidiphilum* genera, with *kdpF* detected exclusively in these acidophiles. The subunit *kdpF* is known to be involved in the stabilization of the potassium transporter (Dorus et al., 2001), suggesting that its presence in the acidophiles may be an adaptation to low pH. In addition, the genomic context of the regulatory genes *kdpDE* in the acidophilic genera is different from the classic organization detected in neutrophiles (Figure 3) (Epstein, 2003). Prediction of HGT events coupled with parsimony analysis shows that the *kdpDE* cluster in these genera is most likely inherited vertically (**Figure 8**). Interestingly, genome rearrangement events have been linked to adaptation in different ecological niches (Darling et al., 2008; Qian and Zhang, 2014; Yan et al., 2018) and we hypothesize that the potential translocation of *kdpD*/*kdpE* in the acidophiles plays a role in low pH adaptation possibly by modifying the expression of the effector proteins KdpFABC in acidic conditions (Ballouz et al., 2010; Cholo et al., 2015). This remains to be tested experimentally.
(iii) We detected a third potassium uptake system (Kup) in acidophiles of *Methylacidimicrobium* and also in several related neutrophiles. However, phylogenetic tree reconstruction shows that Kup of the acidophilic Verrucomicrobia clusters close to Kup of other extreme acidophiles such as *Acidithiobacillus* and *Acidiferrobacter* rather than Kup of neutrophilic relatives (Figure 4), strongly suggesting that it was acquired by HGT, which is supported by HGTector results predicting *Acidithiobacillus* as a putative donor (Supplementary Table 7). As Kup is embedded in the membrane directly facing the physicochemical constraints of the low pH environment, this HGT event could enable the acidophilic methanotrophs to respond to the challenge of low pH. This is consistent with the suggestion that acidophiles are prone to share genes that carry adaptation to their specific econiche (Beard et al., 2021), and supports the proposed role of Kup in some acidic environments (Trchounian and Kobayashi, 1999).

#### Membrane modifications

**Figure 5:**
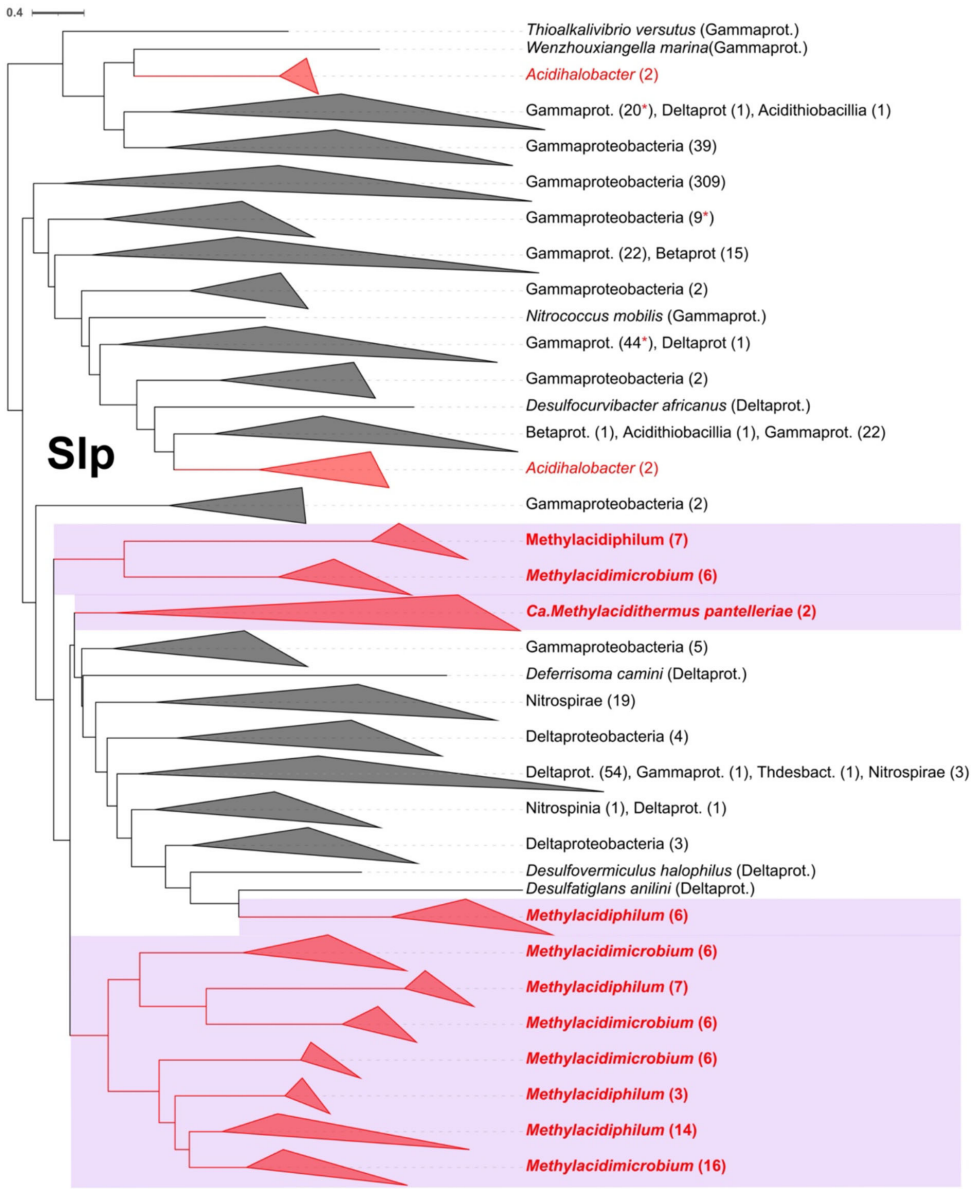
Rooted phylogenetic tree for Slp in Verrucomicrobia acidophiles (violet background) and their best Blast hits. In red are the Slp predicted proteins from acidophiles and in red asterisks are marked the collapsed clades where an acidophile protein sequence is present. The clades were collapsed to class or phylum level when possible, indicating the number of leaves in parenthesis.

The starvation lipoprotein (Slp) is one of the proteins identified involved in acid resistance (Alexander and St John, 1994). Slp was detected exclusively in the acidophilic and not the neutrophilic Verrucomicrobia. The number of copies ranged from a minimum of five in *Methylacidiphilum fumariolicum* to seven in the *Methylacidimicrobium* genus. A rooted phylogenetic tree with the best hits from NCBI for the Slp family was done (Figure 5), showing a close relation with sequences from Gammaproteobacteria, including sequences from the acidophiles Acidihalobacter. The sequences from Verrucomicrobia form different clades suggesting that there are possible different origins for the multiple Slp gained. The lipobox characteristic for this protein was present in 55 of 79 of the orthologs. However, the classic Asp was not found in the +2 position suggesting that Slp is targeted to the outer membrane (Zückert, 2014; Vergara et al., 2020). Extensive gene duplication of *slp* in extreme acidophiles has been noted previously in the genera *Leptospirillum* (Vergara et al., 2020) and *Acidihalobacter* (Boase et al., 2022). Currently, little is known about the mechanism of action of Slp. Transcription analysis in *E. coli* reveals that a gene cluster denominated acid fitness island (AFI) that includes *slp* which is overexpressed under acidic conditions (Tucker et al., 2002) and it is also known that *slp* mutants have a diminished capability to grow in extremely acidophilic pH (Mates et al., 2007), arguing for a link between the presence of *slp* and the capability to grow in low pH conditions.

**Figure 6:**
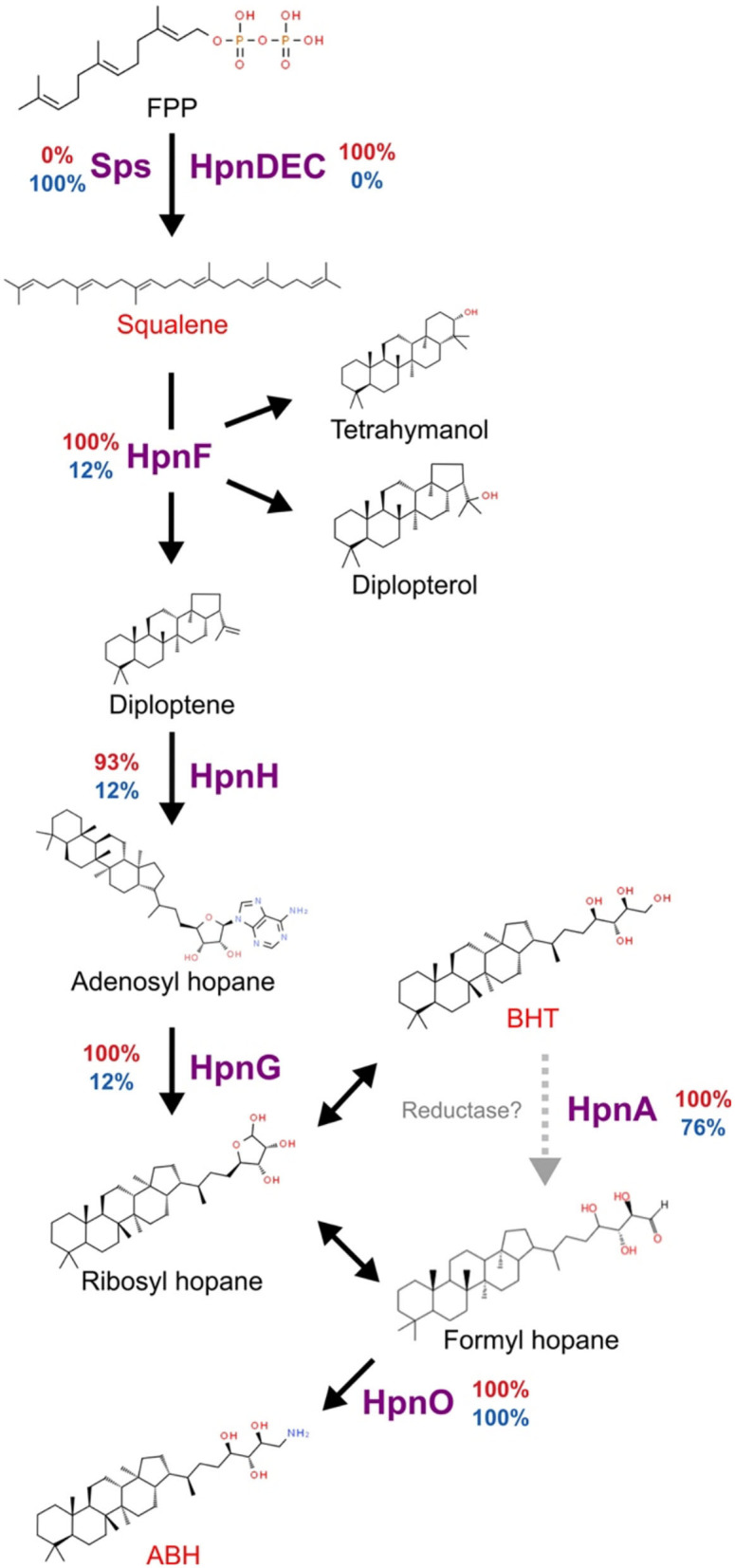
Predicted hopanoid biosynthesis in Verrucomicrobia. The percentage of organisms with each of the proteins is presented at the side of each specific reaction arrow. The percentage in acidophiles is presented in red and neutrophiles in blue. Gray dotted arrow represents a not verified step mediated by HpnA, double arrow indicates spontaneous reactions. FPP, Farnesyl diphosphate; BHT, bacteriohopanetetrol; ABH, Aminobacteriohopanetriol.

One of the main mechanisms identified in acidophilic bacteria to participate in membrane modifications is hopanoids biosynthesis (Belin et al., 2018; Vergara et al., 2020). The genes for squalene biosynthesis, which is the precursor of hopanoid, are present in all acidophiles and 15 of 25 neutrophiles, however, with different genes to complete the process. For instance, in neutrophiles, the squalene synthase (*sps*) gene was identified, while in acidophiles, *hpnCDE* were identified as the enzymes involved in the production of squalene, following a canonical synthesis pathway (Kannenberg and Poralla, 1999). The squalene production genes in several Verrucomicrobia genomes could be explained by their multiple roles as the precursor for sterols and pentacyclic triterpenes in addition to hopanoids, molecules that perform a range of functions on the stabilization of membranes not only, particularly, associated with low pH adaptation (Belin et al., 2018). In contrast, *hpnF* codifying for squalene hopane cyclase and *hpnG* producing the first branched hopanoid molecules were identified in all acidophiles but only in three neutrophiles (*Chthoniobacter flavus* Ellin428, *Terrimicrobium sacchariphilum*, and *Limisphaera ngatamarikiensis*). The hopanoid transporter *hpnN* required for the transport of the cytoplasm synthesized hopanoid molecules to the membranes was identified in the organisms that have *hpnFG* with the addition of two other *Verrucomicrobiales* genomes. The predicted final products in the pathway for acidophiles and *Limisphaera ngatamarikiensis* could be the branched hopene molecules bacteriohopanetetrol (BHT) and aminobacteriohopanetriol (ABH), in contrast to most neutrophiles in Verrucomicrobia, reaching until squalene (Figure 6). HpnO and HpnA were identified in the majority of organisms. The differences in the hopanoid biosynthesis pathway could be key in the development of acid resistance as the branching hopene molecules have been related to adaptation to extremely low pH providing the necessary membrane modifications to reduce the entrance of protons to the cytoplasmic space (Mangold et al., 2013; Belin et al., 2018).

### Acid resistance: Second line of defense

#### Cation antiporters

The sodium–proton exchanger (NaH, *nhe2*) is a membrane protein that transports Na^+^ into the cell and H^+^ out of the cell (antiport). It has been implicated in pH homeostasis in a wide range of species (Masrati et al., 2018). NaH, encoded by *nhe*2, is predicted to occur in the acidophilic methanotrophs *Methylacidiphilum* and *Methylacidimicrobium* where it is present in multiple copies except for *Methylacidiphilum* sp. IT5 which has only one copy (Supplementary Table 3). Phylogenetic analysis indicates that the acidophilic methanotrophs have three distinct clades of NaH suggesting that it may have different origins/functions. The first clade (termed NaH-1) contains sequences from both of the acidophilic genera and is closely related to sequences from Firmicutes and Actinobacteria (Supplementary Figure 1). The second clade (NaH-2) is associated only with *Methylacidiphilum*, branches close to thermophilic microorganisms such as *Thermus*. There is a third clade (NaH-3) that branches close to Proteobacteria, and we speculate that it is not necessarily involved in acid resistance (Supplementary Figure 1). We hypothesize that the NaH restricted to the acidophiles helps in proton export in acidic environments. It remains to be deduced whether it arose by HGT from other acidophiles or by gene duplication from the neutrophilic NaH representative that was present in the ancestral line.

The calcium/proton antiporter (Cah, *chaA*) was identified in all species of the *Methylacidiphilum* genus and is predicted to have been gained by HGT. To study further the distribution of *cha*A, a phylogenetic tree analysis was performed. Two clearly distinct clades could be identified (Supplementary Figure 2). Clade one contains sequences from *Methylacidiphilum* which branch close to, but distinct from, Proteobacteria and *Nitrospira lenta* and also contains the sequences from *Opitutales* and one *Chtoniobacterales*. Clade two does not have any *Methylacidiphilum* representatives but contains sequences from *Chtoniobacterales* and *Verrucomicrobiales*. The distance between Cah from the acidophiles and other members of the Verrucomicrobia is consistent with the idea that it was acquired by HGT supported by HGTector results (Supplementary Table 7). Alternatively, *cha*A in the acidophiles has been subjected to evolutionary pressure to adapt to dealing with low pH environments.

#### Decarboxylation systems

**Figure 7:**
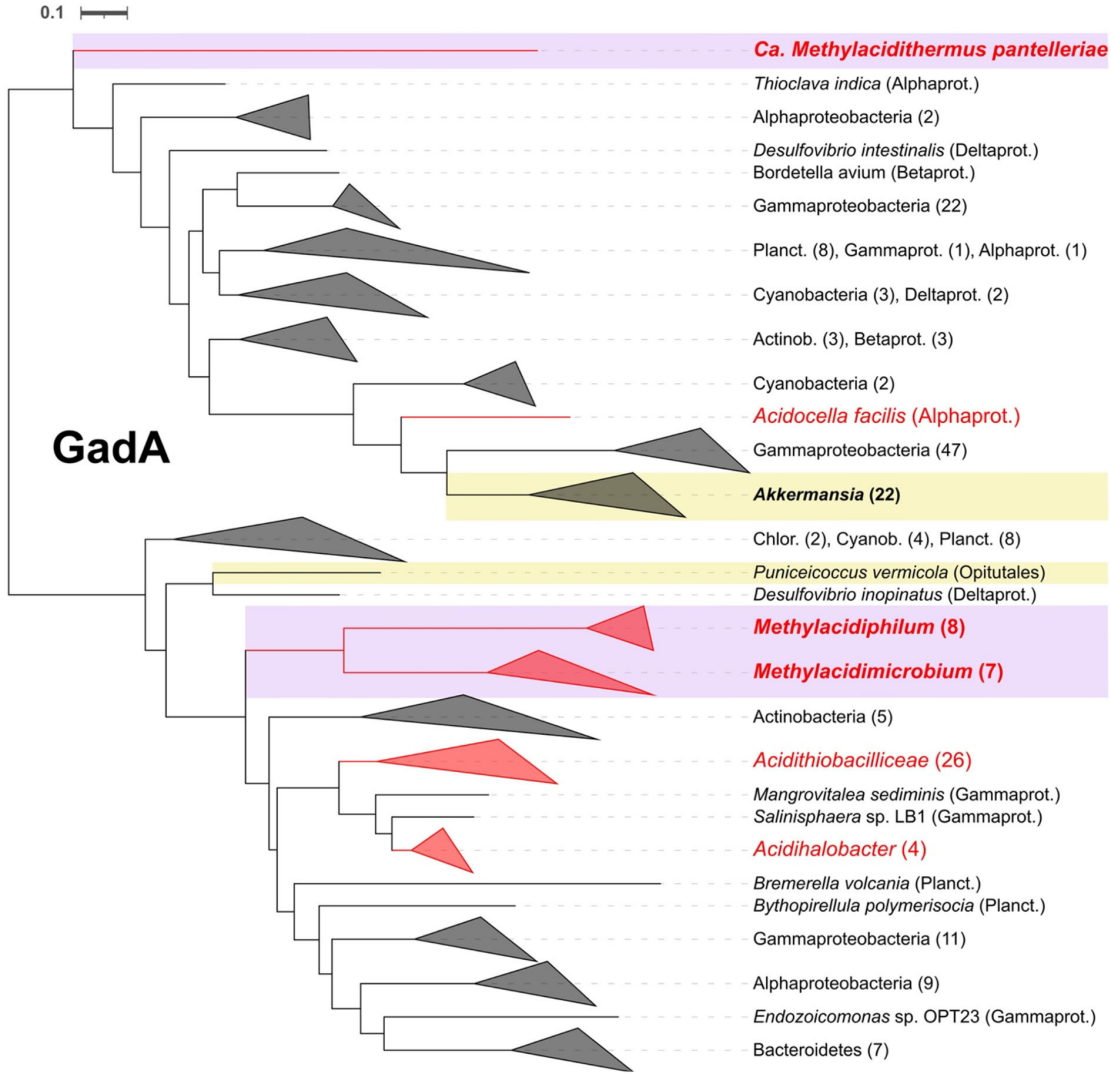
Rooted phylogenetic tree for glutamate decarboxylase GadA. In violet and yellow background are highlighted the acidophiles and neutrophiles from Verrucomicrobia, respectively, with bold letters for the ones present in this study. Collapsed clades to phylum or class level, when possible, with number of leaves between parentheses. Alphaprot, Alphaproteobacteria; Betaprot, Betaproteobacteria; Gammaprot, Gammaproteobacteria; Deltaprot, Deltaproteobacteria; Planct, Planctomycetes; Acctinob, Actinobacteria; Chlor, Chloroflexi; Cyanob, Cyanobacteria.

A GadA (glutamate decarboxylase) decarboxylation system, potentially involved in acid resistance (Bearson et al., 1997; Foster, 2004), was identified in both acidophilic and neutrophilic Verrucomicrobia. GadA was identified in all acidophilic species and only in two neutrophiles (Supplementary Table 3). Phylogenetic tree analysis of GadA (Figure 7) indicates that its closest relatives include multiple examples from other acidophiles of the genus *Acidithiobacillus*, suggesting that it was obtained by HGT. GadA has been shown to enhance the ability of bacteria to cope with extremely low pH (Damiano et al., 2015) and to function in *E. coli* when stressed at low pH (Foster, 2004). GadA works in conjunction with the antiporter system for the export of protonated products. Two conserved potential antiporter systems, GadC and AdiC (Hou et al., 2008; Kruse et al., 2019), were detected in methanotrophs but it remains to be demonstrated experimentally that they function in collaboration with the decarboxylation systems.

#### ATPase F_0_-F_1_

ATPase F_0_-F_1_ is a system used for both the generation of ATP and the extrusion of protons in highly acidic conditions (Sun, 2016). Two different ATPase F_0_-F_1_ clusters were identified in the Verrucomicrobia genomes. The first, conserved across all organisms exhibit a genetic structure that follows the canonical organization *atpBEFHAGDC* of an F-type ATPase involved in ATP synthesis. The second cluster is only present in *Methylacidiphilum* and *Methylacidimicrobium* and has the organization *atpDCZBEFAG*, resembling that of an N-type ATPase (Supplementary Figure 3A) (Schulz et al., 2017). The motif ESLxxY was detected in subunit *atp*E (Supplementary Figure 3B), which is highly conserved in acidophiles and has been suggested to be involved in proton extrusion as a mechanism for acid resistance (Foster, 2004; Sun et al., 2012).

#### Intracellular buffering

Intracellular buffering is another mechanism commonly used by cells for internal pH homeostasis (Slonczewski et al., 2009). One of the pathways predicted in both acidophilic and neutrophilic Verrucomicrobia is the arginine/agmatine deiminase pathway. This pathway includes the participation of the arginine decarboxylase (SpeA or AdiA) and two additional proteins: agmatine deiminase (AguA) and N-carbamoylputrescine amidohydrolase (AguB). The synteny of *agu*AB is highly conserved in Verrucomicrobia but their genomic context differs between acidophilic and neutrophilic representatives. For example, all genomes from the *Methylacidimicrobium* clade have a syntenic downstream region that contains the hopanoid transporter *hpnN* and the *Methylacidimicrobium* and *Methylacidiphilum* genera have an upstream *slp* (Supplementary Figure 4). Since we have detected a possible relationship between *slp* and *hpn* with acid resistance, it is possible that *aguAB* are also associated with pH homeostasis.

Another mechanism commonly used for intracellular buffering is the phosphate uptake system (*pstABCS*) (Hutkins and Nannen, 1993; Peng et al., 2017). PstABC was identified in conserved orthogroups across acidophilic and neutrophilic Verrucomicrobia and we suggest that this gene cluster was acquired by vertical inheritance. In contrast, the gene potentially encoding the phosphate binding subunit PstS was identified only in three neutrophiles but was conserved across all acidophiles. To explore this in further detail, a phylogenetic tree was constructed. This tree shows a clear separation of *pst*S between the acidic and neutrophilic clades (Supplementary Figure 5) suggesting that its acquisition was by HGT.

Carbonic anhydrase (Can) has also been implicated in intracellular buffering by a proton-consuming reaction and is upregulated under acidic conditions in *Helicobacter pylori* (Bury-Moné et al., 2008). Ca was predicted in both acidophilic and neutrophilic Verrucomicrobia but could be separated by phylogenetic analysis into two orthogroups corresponding, respectively, to acidophilic and neutrophilic Verrucomicrobia, with the caveat that Ca. *Methylacidithermus* and two organisms from *Methylacidimicrobium* have copies of both orthogroups (Supplementary Table 3). Using bioinformatic approaches, we could not detect functional differences between the two orthogroups and their potential role in pH homeostasis in the Verrucomicrobia remains to be elucidated.

### Evolutionary trajectory of acid resistance genes

**Figure 8:**
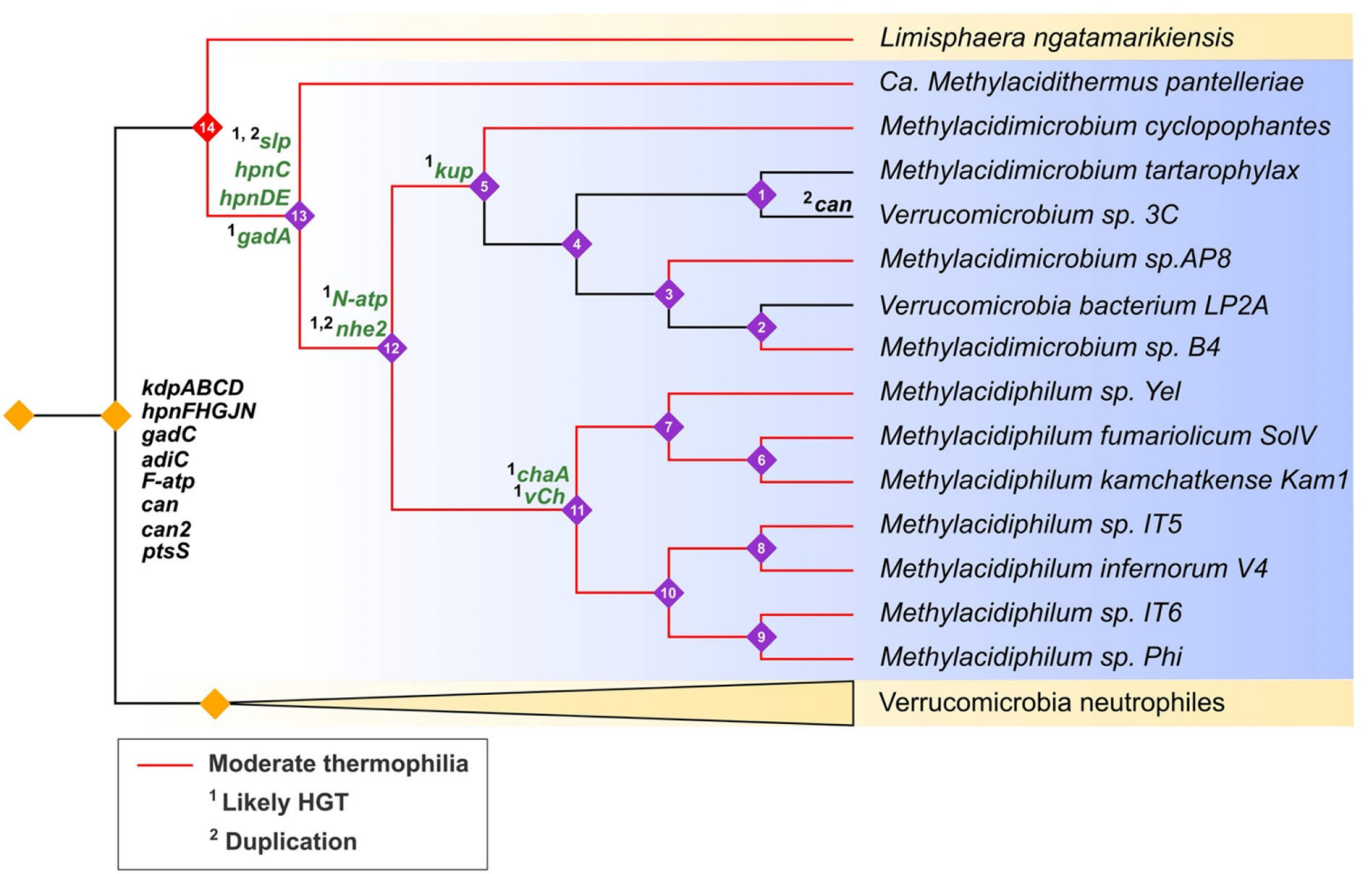
Hypothesized evolutionary trajectories of genes predicted to be involved in acid resistance in *Verrucomicrobia* (cladogram). Acidophiles = blue background with purple numbered nodes; neutrophiles = yellow background with unnumbered orange nodes. Clades with red branches are moderate thermophiles and black branches are mesophiles. Genes in black are inferred to be inherited by vertical descent, genes in green are predicted to be inherited by ^1^HGT or ^2^gene duplications. Gene events were predicted by Count with Dollo parsimony, HGTector, and phylogenetic analyses.

To gain insight into the evolutionary history of the acidophilic Verrucomicrobia, genes predicted to be involved in acid resistance were mapped onto a cladogram derived from the maximum likelihood tree of Verrucomicrobia. Gene gains (HGT and gene duplications) were annotated using Dollo parsimony analysis and HGT prediction (Figure 8). The cladogram is consistent with the idea that the acidophiles (blue background, Figure 8) evolved from a common ancestor shared with the neutrophiles (yellow background, Figure 8). Interestingly, the early evolution of the acidophiles appears to be concurrent with the development of moderate thermophilia (growth ~50°C) (red branches, Figure 8). The inferred neutrophilic ancestor already had several genes involved in acid resistance including *kdp*ABCD and various Hpn modifications (black genes, Figure 8). We speculate that these could have provided an advantage for growth in moderately low pH conditions (pH 4-6). However, for growth in extremely low pH, various genes were gained such as *slp, N-atp, hpnCDE*, and *nhe* (green highlighted gene, Figure 8). It is proposed that several of these genes were gained by HGT from other acidophilic lineages as discussed above; other genes such as *slp* are predicted to have been gained by HGT followed by rampant gene duplication.

Reduction of genome size was also observed in the proposed evolutionary trajectory with an average genome size of the neutrophiles of 5.3 Mb, whereas it was 2.37 Mb in the acidophilic clades (Supplementary Table 1). Genome reduction has been proposed to be an adaptive mechanism to reduce energy costs involved in replication and translation in nutrient-scarce environments (Sowell et al., 2009) and under acid stress (Cortez et al., 2022).

## Model

**Figure 9:**
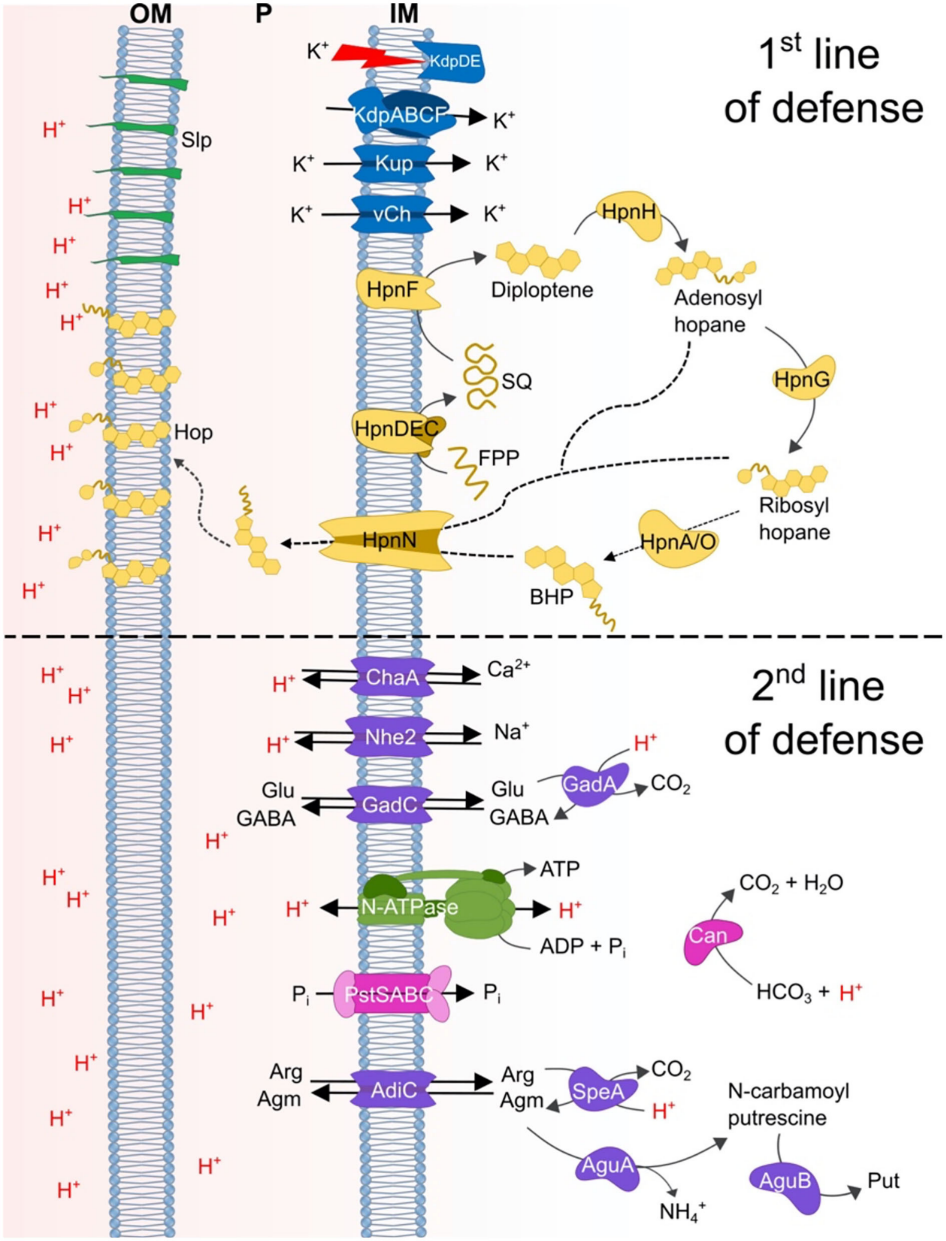
Model of the proposed acid resistance mechanisms in Verrucomicrobia acidophiles. Top section shows the predicted first line of defense mechanisms: blue = potassium/H+ exchange systems involved in the generation of a reversed membrane potential and the membrane systems Slp and Hpn; green, Slp; yellow, proteins associated with hopanoid biosynthesis. Bottom section shows the proposed second line of defense mechanisms: purple, decarboxylation and antiporter systems; green, N-type ATP synthase; pink, Pst transporter and carbonic anhydrase.

A model of genes and mechanisms predicted to be involved in acid resistance in extreme acidophiles belonging to the Verrucomicrobia group is shown in Figure 9. The model incorporates the concept of first and second lines of defense, where the first line of defense consists of mechanisms that are used to impede the entry of protons into the cell. These are principally, but not exclusively, located in the cell membrane. Whereas second line of defense mechanisms are mainly involved in the neutralization or expulsion of protons that have either escaped the first line of defense and have entered the cell or have been produced within the cell.

## Data availability statement

The original contributions presented in the study are included in the article/Supplementary material, further inquiries can be directed to the corresponding author/s.

## Author contributions

GN and DH conceived and designed the research. GN performed the research. GN, EV, and DH analyzed the data and wrote the manuscript. All authors participated in the writing and approval of the final manuscript.

## Funding

This work was supported by FONDECYT 1181717 (DH), Centro Ciencia & Vida, and FB210008 Financiamiento Basal para Centros Científicos y Tecnológicos de Excelencia de ANID.

## Conflict of interest

The authors declare that the research was conducted in the absence of any commercial or financial relationships that could be construed as a potential conflict of interest.

## Publisher's note

All claims expressed in this article are solely those of the authors and do not necessarily represent those of their affiliated organizations, or those of the publisher, the editors and the reviewers. Any product that may be evaluated in this article, or claim that may be made by its manufacturer, is not guaranteed or endorsed by the publisher.
