# Supplementary material for: Genome-guided prediction of acid resistance mechanisms in acidophilic methanotrophs of phylogenetically deep-rooted Verrucomicrobia isolated from geothermal environments
Source: Front Microbiol. 2022 Sep 23;13:900531. doi: 10.3389/fmicb.2022.900531 (PMC9543262; doi:10.3389/fmicb.2022.900531)
Supplement: Supplementary file 1 [file Data_Sheet_1.PDF]

Supplementary Material

1. Supplementary Figures

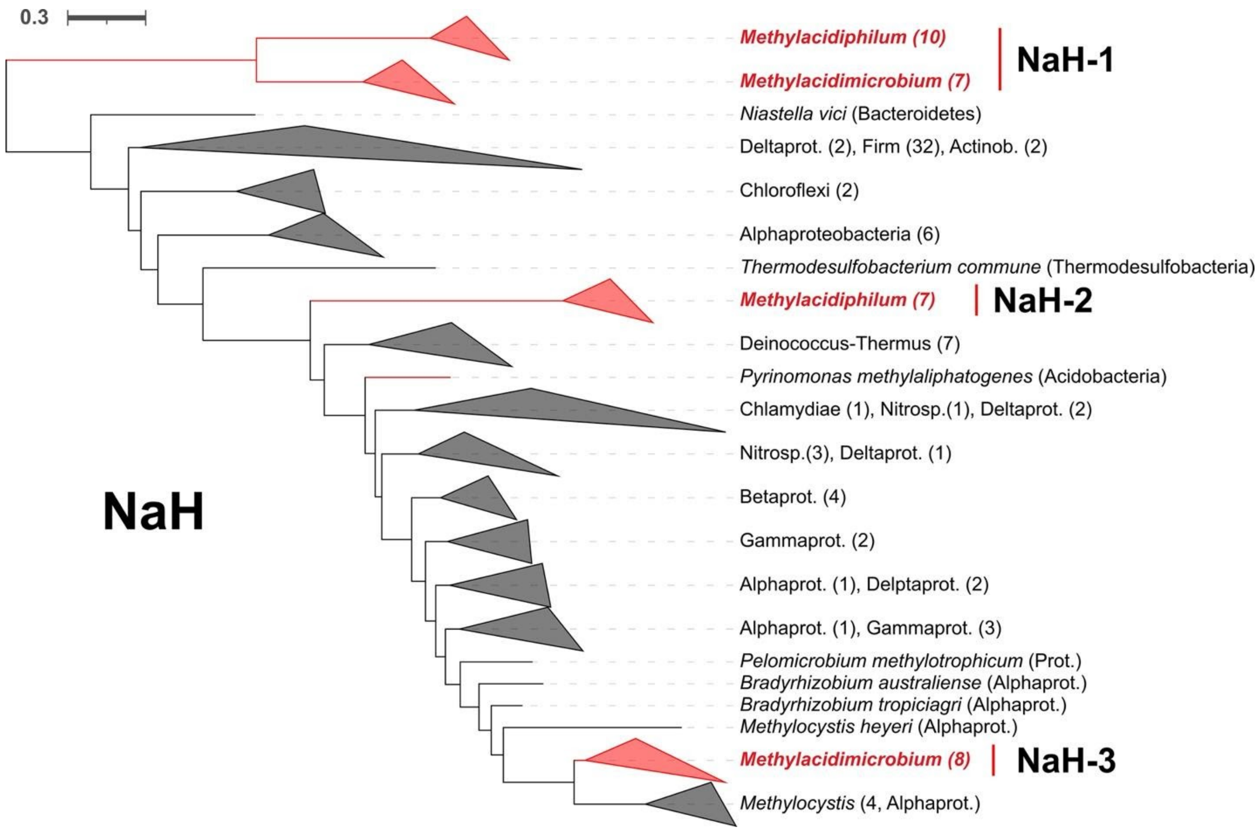

Supplementary Figure 1. Rooted phylogenetic tree of the Sodium/proton antiporter NaH sequences from Verrucomicrobia and their best hits from NCBI. In red are marked the clades of Verrucomicrobia acidophiles. The clades were collapsed, when possible, to phylum or class taxonomic level indicating in parenthesis the number of leaves in each.

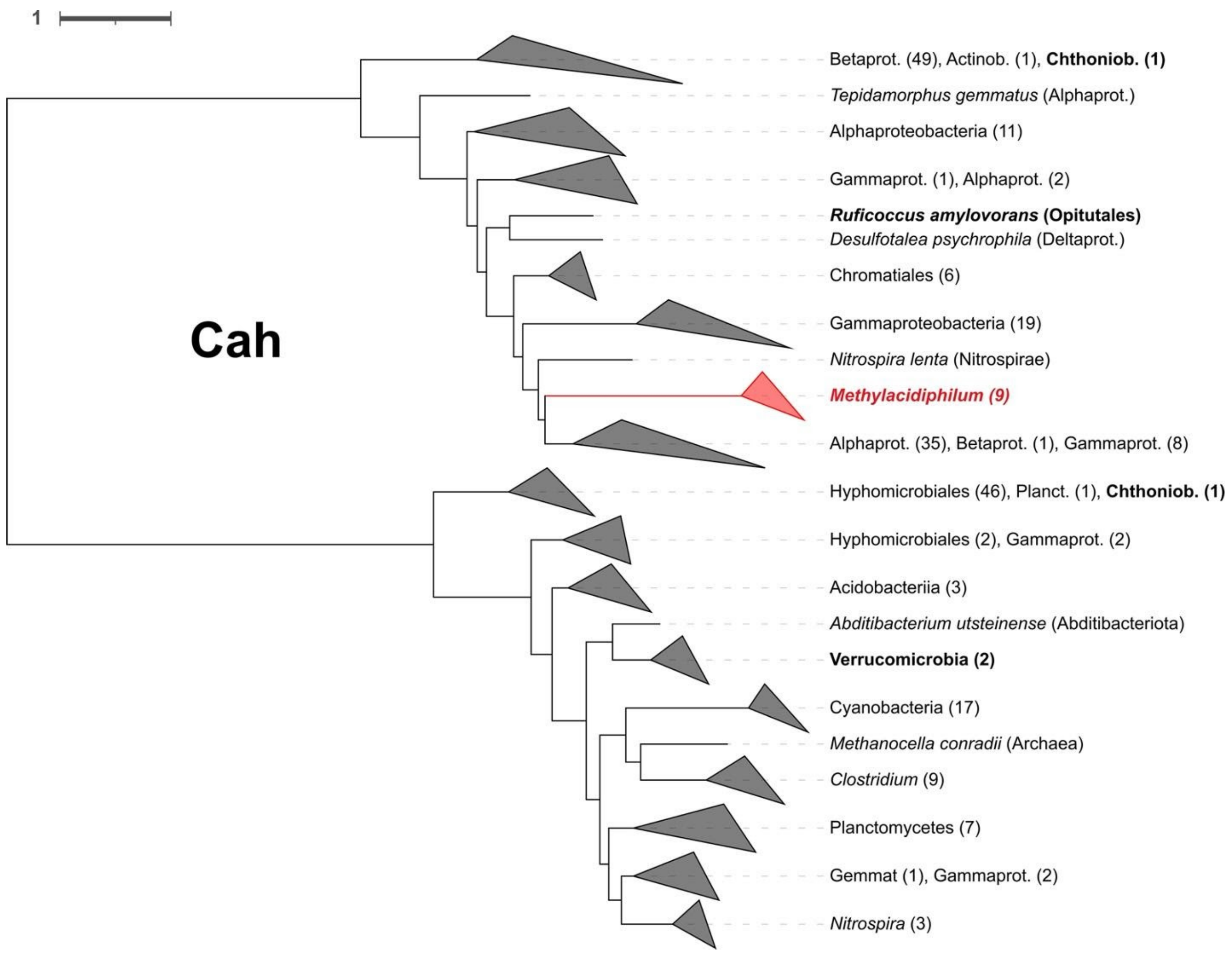

Supplementary Figure 2. Rooted phylogenetic tree of Calcium/proton antiporter Cah from Verrucomicrobia and their best hits. In red are marked the clades of Verrucomicrobia acidophiles. The clades were collapsed when possible, to phylum or class taxonomic level, indicating in parenthesis the number of leaves in each.

A.

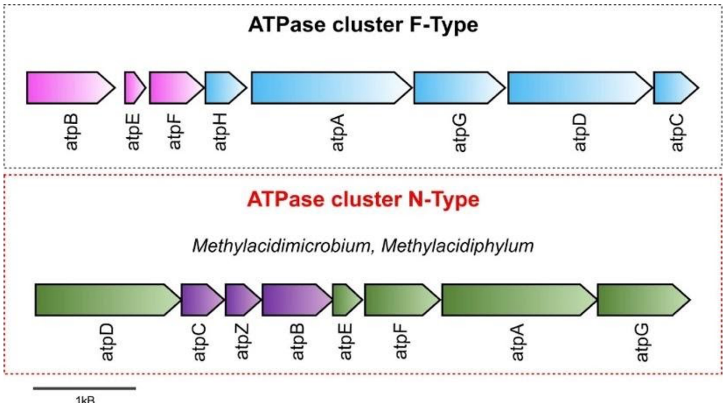

B.

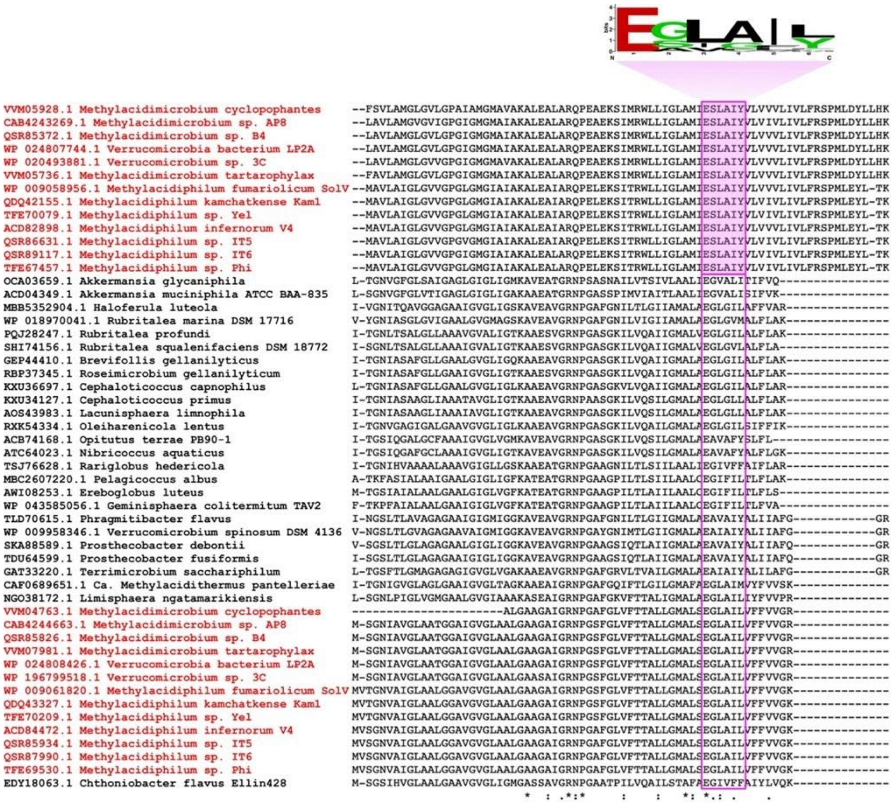

Supplementary Figure 3. **A.** ATPase F-type genes in all genomes of Verrucomicrobia (top) and ATPase N-type gene cluster present only in *Methylacidimicrobium* and *Methylacidiphilum* acidophilic genera **B.** MSA of AtpE sequence (C-term), in purple square is marked the conserved motif ESLxxY which helps to identify the N-type ATPase.

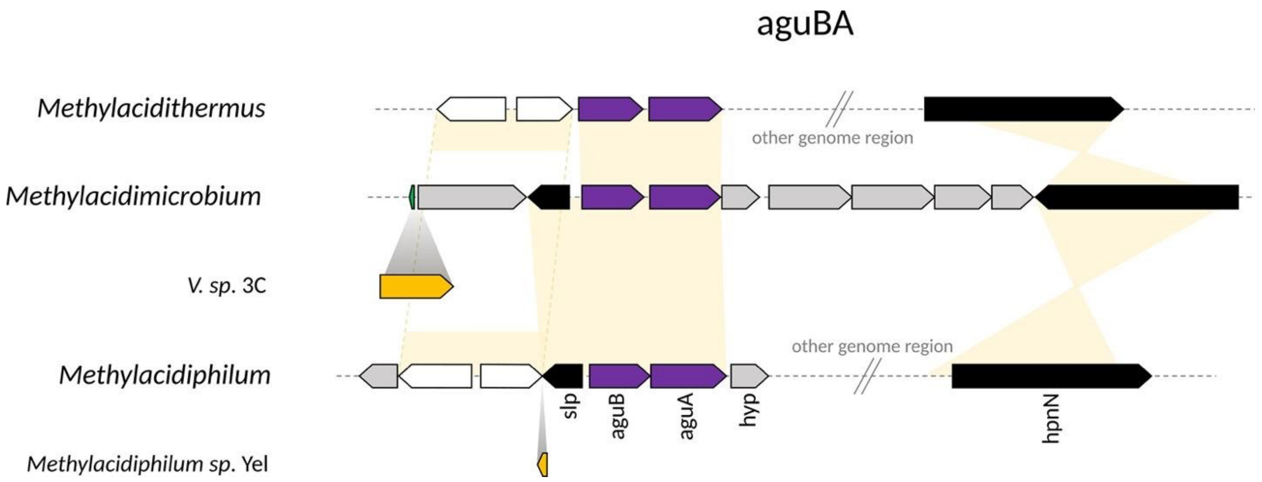

Supplementary Figure 4. *aguBA* gene context in Verrucomicrobia acidophiles. In purple are the *aguBA* genes, in black are other acid resistance related genes, in white are genes conserved between non-mesophiles genera, in yellow are mobile elements (transposases) genes, in green it is a tRNA-His and in gray are other genes. Synteny is marked with light yellow shadows connecting blocks.

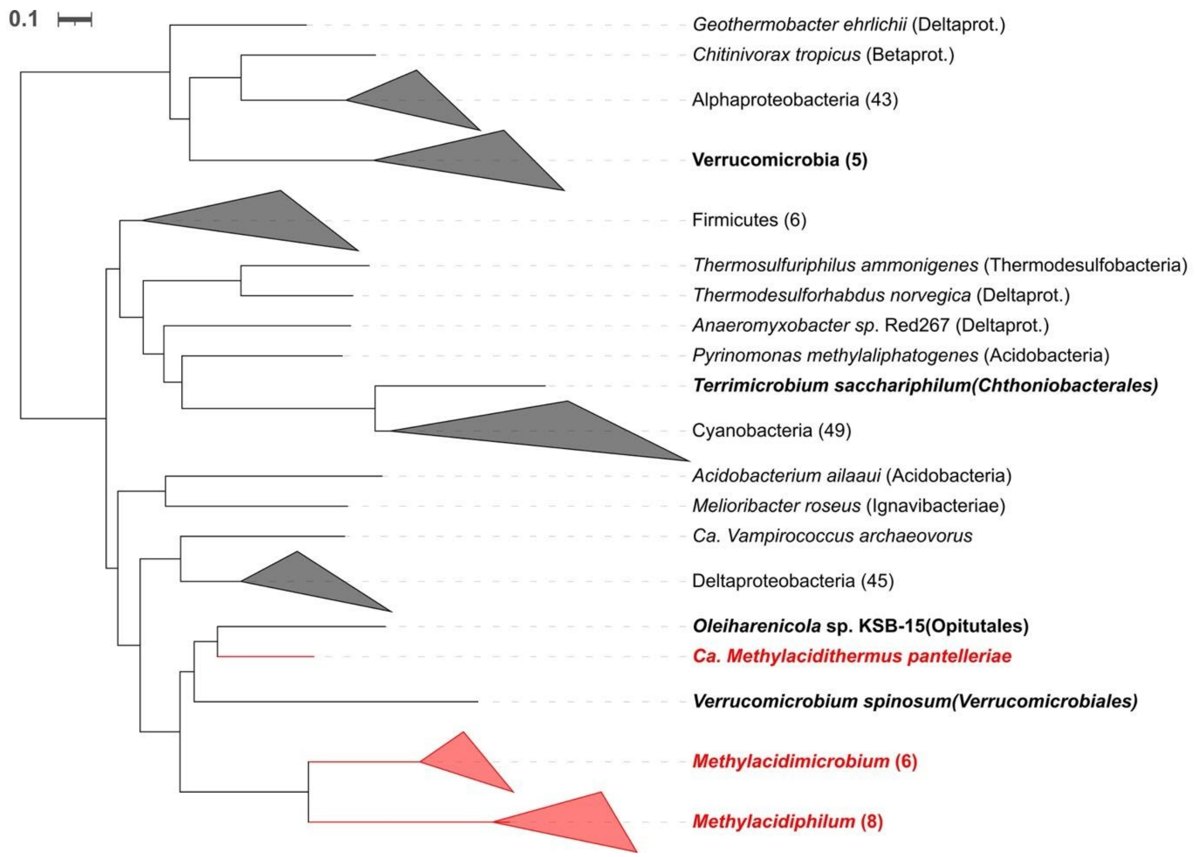

Supplementary Figure 5. Rooted phylogenetic tree of *pstS*. In red are marked the clades of Verrucomicrobia acidophiles. The clades were collapsed when possible, to phylum or class taxonomic level, indicating in parenthesis the number of leaves in each.
